# Nasal eosinophilia in pediatric non-allergic rhinitis: correlation with clinical severity scales

**DOI:** 10.1186/s12887-026-07197-4

**Published:** 2026-06-27

**Authors:** Hüseyin Günizi, Gözde Orhan Kubat, Özlem Ceren Günizi, Ergün Sevil, Emre Yıldırım, Anıl Eren

**Affiliations:** 1 Department of Otolaryngology, Medical School, Akdeniz Üniversity, Antalya, Turkey; 2 Department of Otolaryngology, Medical School, Alanya Alaaddin Keykubat Üniversity, Antalya, Turkey; 3 Department of Medical Pathology, Medical School, Akdeniz Üniversity, Antalya, Turkey; 4 Clinic of Otorhinolaryngology and Head and Neck Surgery, Private Clinic, Alanya, Turkey

**Keywords:** Non-allergic rhinitis, NARES, Nasal eosinophilia, Pediatric, ARIA, PRQLQ, Nasal cytology, Quality of life

## Abstract

**Background:**

Non-allergic rhinitis syndrome (NAR) is a chronic rhinitis characterized by the significant absence of an allergy history, negative skin prick test results, and normal serum IgE levels. Nasal cytology is a valuable diagnostic method that enables qualitative and quantitative assessment of inflammatory cells — including eosinophils, neutrophils, mast cells, and lymphocytes — in the nasal mucosa. This study aimed to evaluate the levels of nasal eosinophilia in a pediatric NAR population and to evaluate the correlation of this local inflammatory biomarker with clinical severity scales such as ARIA and PRQLQ.

**Methods:**

This prospective, cross-sectional study included 103 children aged 5–18 years: 53 with NAR and 50 healthy controls. Symptom duration and severity were classified according to ARIA 2019 criteria. The Paediatric Rhinitis Quality of Life Questionnaire (PRQLQ) was used for quality of life assessment. Nasal cytology specimens were collected by nasal swab from the middle meatus and the eosinophil percentage was calculated by counting a total of 100 cells in the area of highest cell density.

**Results:**

A total of 103 children were enrolled: 53 NAR patients and 50 healthy controls. Median age was 10 (8–13) years in the study group and 11 (8–14) years in the control group. Family history of allergy was significantly higher in the study group (30.2%) compared to controls (12.0%) (*p* = 0.024). Median nasal eosinophil level was 7.0 (3.5–15.5) in the study group and 0 (0–3.0) in controls; the difference was statistically significant (*p* < 0.001). The nasal eosinophil cut-off value was determined as 3.5%. No significant difference was found between nasal eosinophil groups (< 3.5% and ≥ 3.5%) and any PRQLQ subscale or total score (*p* > 0.05).

**Conclusions:**

Nasal cytology may serve as a simple, non-invasive diagnostic tool to identify NAR subtypes and to determine clinical severity in pediatric patients.

## Background

Rhinitis is an inflammatory disease affecting the nasal mucosa, presenting with clinical manifestations such as rhinorrhea, nasal congestion, sneezing, and pruritus. It can be classified into three main categories: allergic rhinitis (AR), infectious rhinitis, and non-allergic rhinitis (NAR) [1]. NAR is classified into four distinct subtypes based on the predominant inflammatory cell profile in the nasal mucosa: eosinophilic, neutrophilic, mixed-type, and pauci-inflammatory [2]. Accurate identification of the predominant inflammatory phenotype within the NAR spectrum is important for individualized treatment, as different cell types respond differently to therapies. Due to the insufficient clinical and pathological definition of NAR subtypes, this condition is underrecognized by clinicians and its epidemiological burden in the pediatric age group is underestimated. The exact prevalence of NAR in the pediatric population is unknown; however, the comparative prevalence of NAR to AR has been reported to be at least 1:3–4 [3, 4]. Although NAR is underestimated compared to AR, approximately 200 million people are affected by this disease [5]. In clinical practice these forms are often overlapping, and approximately 15–20% of patients may have both AR and NAR [6]. Studies show that serum total IgE levels are comparable to control groups, which explains the ‘non-allergic’ nature of the syndrome [7]. Eosinophils are the primary cell type involved in the pathogenesis of both NAR and AR [8]. It has been demonstrated that eosinophils may be present in nasal smears of atopic children before the detection of specific serum IgE, and that they are strongly associated with disease severity and the development of airway hyperreactivity [9, 10]. It has been suggested that the level of nasal eosinophilia may be associated with response to medical treatment. Different types of NAR are characterized by different prognoses and responses to medical treatments. Although recognition of these conditions in children is of epidemiological importance, they are often misdiagnosed and underestimated because they do not conform to any rhinitis subgroup according to formal criteria. These clinical similarities increase the need for objective tests — such as nasal cytology and/or local IgE assessment or nasal provocation tests — to help distinguish the inflammatory structure involved in pathogenesis [11, 12]. Since various NAR forms present with similar symptoms, history and standard tests are inadequate for differential diagnosis. Nasal cytological examination comes to the fore as a cost-effective and efficient method for phenotyping NAR patients [13]. The procedure is non-invasive, low-cost, and easily repeatable. Specimens obtained by swabbing, scraping, brushing, lavage, or aspiration are typically stained with Wright-Giemsa or May-Grünwald-Giemsa. However, nasal cytology (NC) alone may not be sufficient to differentiate cases of local allergic rhinitis (LAR); in this case, the gold standard is the nasal provocation test.

The Paediatric Rhinitis Quality of Life Questionnaire (PRQLQ) and the Allergic Rhinitis and its Impact on Asthma (ARIA) score — validated clinical scales commonly used for assessing symptom severity and quality of life — are standardized measures that allow monitoring of treatment response. Literature has demonstrated that PRQLQ successfully measures quality of life including nasal and ocular symptoms as well as practical problems and activity limitations, and that it reflects clinical changes [14, 15]. The negative impact of moderate-to-severe rhinitis according to ARIA criteria on children’s sleep patterns, academic performance, and social activities has been clearly demonstrated through quality of life scales [16]. The use of these standardized questionnaires enables the elucidation of the multidimensional effects of rhinitis on quality of life and performance in the pediatric population.

This study aimed to evaluate the levels of nasal eosinophilia in a pediatric NAR population. Furthermore, we aimed to evaluate the correlation of this local inflammatory biomarker with clinical severity scales such as ARIA and PRQLQ, thereby contributing to accurate diagnosis and treatment planning in the clinical management of non-allergic rhinitis.

## Methods

This prospective, cross-sectional study was conducted between May 2023 and March 2024 at the Ear Nose and Throat outpatient clinic of Alanya Training and Research Hospital of Alanya Alaaddin Keykubat University Faculty of Medicine, with approval from the Clinical Research Ethics Committee (date: 27/09/2023, decision no: 13 − 02). Written informed consent was obtained from the parents of all participants. A total of 103 children aged 5–18 years were enrolled: 53 with NAR and 50 healthy children without rhinitis symptoms. The control group consisted of 50 healthy, age- and gender-matched children with no rhinitis symptoms or history of atopic disease, negative serum IgE and skin prick tests, no active infection, and presenting with non-respiratory complaints. Inclusion criteria were: chronic rhinitis diagnosis for at least one year, no rhinitis treatment in the past month, no active acute upper respiratory tract infection, and normal serum total IgE levels (defined as < 100 IU/mL) alongside negative skin prick test results. Exclusion criteria included: chronic comorbidities, history of malignancy, systemic inflammatory disease or immunodeficiency, and use of rhinitis treatment in the past month. Participants’ height, weight, gestational age, birth weight, neonatal intensive care history, household smoking exposure, daycare history, family atopy and sociodemographic data were recorded. Symptom duration and severity were classified according to ARIA 2019 criteria. Quality of life was assessed using the PRQLQ.

Nasal cytology specimens were collected by sterile cotton swab from the middle meatus and spread onto glass slides. Slides were dried at room temperature, fixed with alcohol, and stained with Giemsa stain. Cytological evaluation was performed under light microscopy at ×400 magnification. The eosinophil percentage was calculated by counting a total of 100 cells in the area of highest cell density. All evaluations were performed by a single pathologist who was blinded to the clinical data and study groups of the patients.

Data analysis was performed using SPSS 30.0 software. Distribution of variables was assessed using Kolmogorov-Smirnov and Shapiro-Wilk tests and visual graphs; all data except NE% and LYM%, which showed normal distribution, were analyzed with non-parametric tests. Student’s t-test, Mann-Whitney U test, and Chi-square test were used for intergroup comparisons. A multivariate logistic regression analysis was applied to identify independent predictors of NAR. The model included the nasal eosinophil ratio, age, gender, and family history of allergy as independent variables. The diagnostic accuracy of the nasal eosinophil ratio was determined by Receiver operating characteristic (ROC) curve analysis; with a cut-off value of 3.5%, sensitivity of 75.5% and specificity of 86.0% (Youden index: 0.615) were achieved, and the study group was divided into two subgroups (< 3.5% and ≥ 3.5%). Statistical significance threshold was set at *p* < 0.05.

## Results

A total of 103 children were enrolled: 53 NAR patients and 50 healthy controls (Table 1). Median age was 10 (8–13) years in the study group and 11 (8–14) years in the control group. No statistically significant differences were found between groups in terms of age, height, or weight. Family history of allergy was 30.2% in the study group and 12.0% in the control group, and this difference was statistically significant (*p* = 0.024). No significant differences were found between groups in terms of sex, intensive care need, hyperbilirubinemia, breastfeeding history, smoking exposure, or presence of siblings (*p* > 0.05). No significant differences were identified in maternal or paternal education level comparisons (*p* = 0.094 and *p* = 0.330, respectively).

**Table 1:** Demographic and clinical characteristics of the groups

Variable	Study Group(*n* = 53)Median (IQR)	Control Group(*n* = 50)Median (IQR)	*p* value
Age (years)	10 (8–13)	11 (8–14)	0.428
Height (cm)	135 (125–153.5)	137 (120.75–158)	0.670
Weight (kg)	31 (25–50)	34 (27.75–54.25)	0.291
Gestational age (weeks)	38 (36–39)	38 (37–39)	0.127
Birth weight (g)	3120 (2800–3300)	3225 (2942.5–3520)	0.075
IgE (IU/mL)	28.8 (17.9–60.35)	33.6 (16.12–65.03)	0.843
CRP (mg/L)	1.4 (0.7–3.05)	1.2 (0.7–2.53)	0.537
Family allergy history — yes, n (%)	16 (30.2%)	6 (12.0%)	0.024*
Sex — female, n (%)	32 (60.4%)	28 (56.0%)	0.653

*IQR *Interquartile range,* NLR *Neutrophil-to-lymphocyte ratio,* ELR *Eosinophil-to-lymphocyte ratio. *p < 0.05

**Fig. 1:**
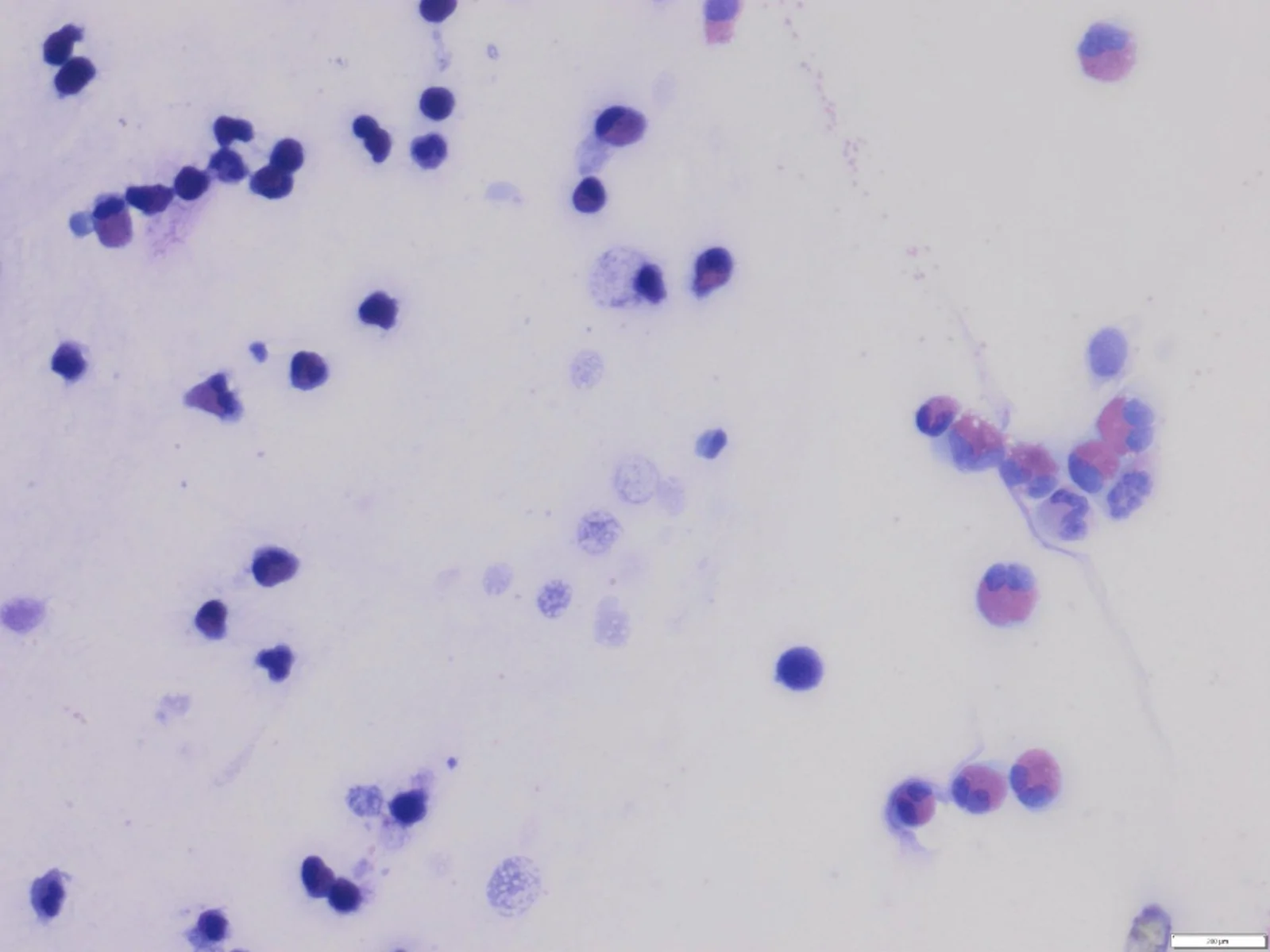
Nasal cytology of NARES. Eosinophil cells are indicated by arrows. Giemsa staining. Magnification ×400

**Fig. 2:**
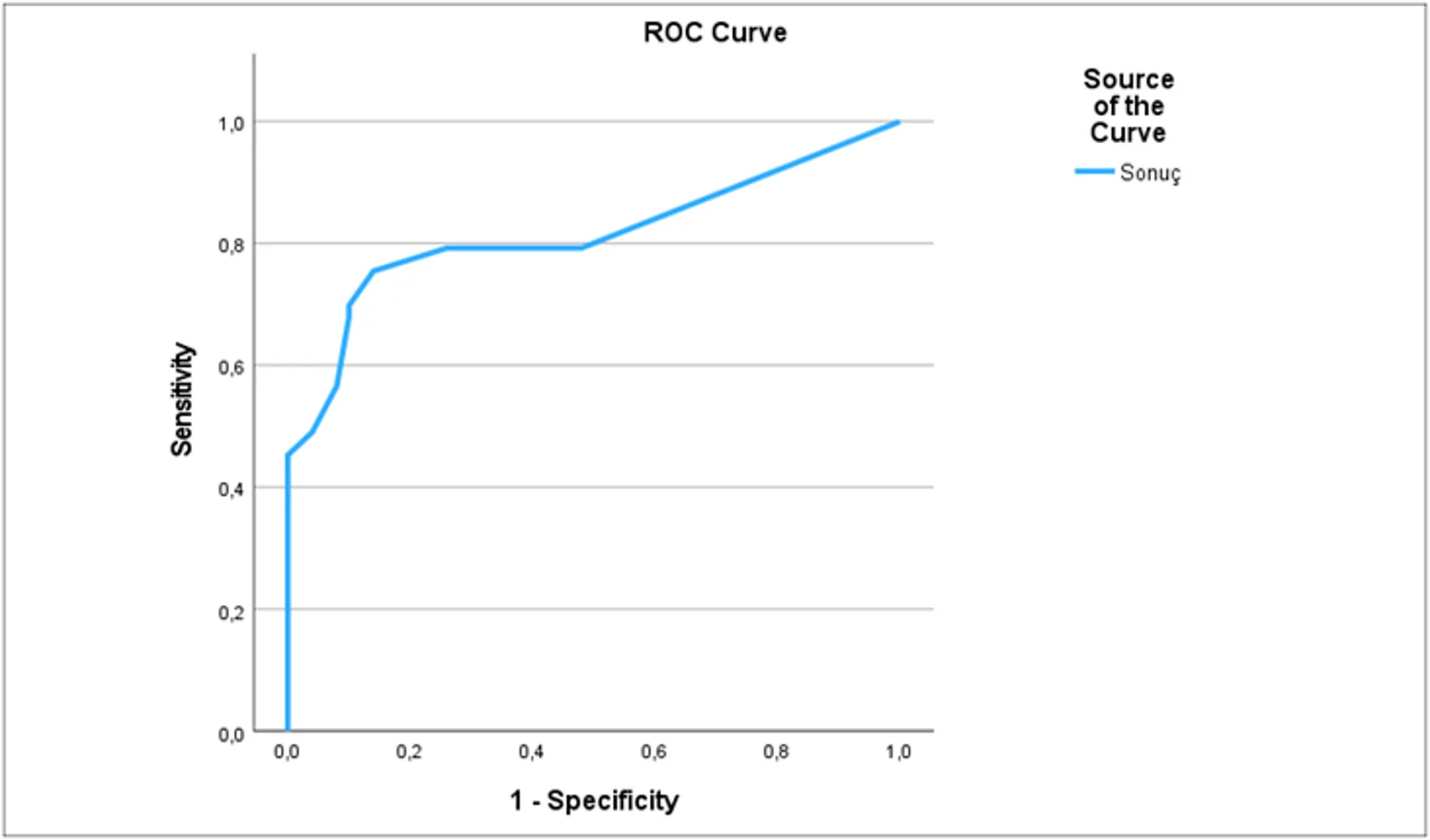
ROC curve analysis of nasal eosinophilia. AUC = 0.815; cut-off 3.5%; sensitivity 75.5%; specificity 86.0%; Youden index 0.615

Since the nasal eosinophil ratio did not follow a normal distribution, the median value was used. Median nasal eosinophil level was 7.0 (3.5–15.5) in the study group and 0 (0–3.0) in the control group; the difference between groups was statistically highly significant (*p* < 0.001) (Fig. 1). The nasal eosinophil ratio was evaluated by ROC curve analysis. The area under the curve (AUC) was calculated as 0.815. At the 3.5% cut-off value, sensitivity was 75.5%, specificity was 86.0%, and the Youden index was 0.615 (Fig. 2). No statistically significant independent predictor other than the nasal eosinophil ratio was identified by logistic regression analysis. The multivariate logistic regression analysis confirmed that only the nasal eosinophil ratio was a statistically significant independent predictor of NAR (OR = 1.406, 95% CI: 1.211–1.634, *p* < 0.001). Total PRQLQ score was calculated as 38.02 ± 21.25 (Table 2). When nasal eosinophil groups (< 3.5% and ≥ 3.5%) were compared, no statistically significant difference was found between any PRQLQ subscale or total score (*p* > 0.05). According to ARIA 2019 criteria, 88.7% (*n* = 47) of patients were classified as intermittent and 11.3% (*n* = 6) as persistent phenotype. In severity classification, 34.0% (*n* = 18) were in the mild group and 66.0% (*n* = 35) in the moderate/severe group. The distribution of the four subgroups was as follows: intermittent-mild 32.1% (*n* = 17), intermittent-moderate/severe 56.6% (*n* = 30), persistent-mild 1.9% (*n* = 1), and persistent-moderate/severe 9.4% (*n* = 5).

**Table 2:** Comparison of PRQLQ subscale and total scores by nasal eosinophil groups

PRQLQ Subscale	Mean ± SD(Entire study group)	*p* value(Eosinophil group comparison)
Nasal symptoms	10.17 ± 4.61	0.754
Ocular symptoms	4.06 ± 3.88	0.975
Practical problems	7.34 ± 6.05	0.375
Other symptoms	9.92 ± 7.27	0.772
Activity limitation	5.17 ± 4.39	0.653
Total score	38.02 ± 21.25	0.694

*SD* standard deviation. Comparison: between < 3.5% and ≥ 3.5% nasal eosinophil groups

The neutrophil-to-lymphocyte ratio was significantly higher in the moderate/severe group [mean 1.76 ± 0.89] compared to the mild group [mean 1.29 ± 0.53] (*p* = 0.045). No significant differences were found between groups for all other parameters (IgE, CRP, neutrophil count, lymphocyte count, ELR, quality of life scores, symptom score) (*p* > 0.05).

Serum IgE level was significantly higher in the persistent group (median 60.7 (IQR: 27.0–101.0) IU/mL) compared to the intermittent group (median 26.0 (IQR: 17.7–53.3) IU/mL) (*p* = 0.026). No significant differences were found between the two groups for other laboratory parameters, demographic data, and symptom scores (*p* > 0.05). Although ARIA 2019 symptom score (mean 5.8 ± 3.25) and age at daycare entry (mean 4.0 ± 2.10 years) were higher in the persistent group compared to the intermittent group (3.5 ± 2.20 and 2.3 ± 2.40 years, respectively), these differences did not reach statistical significance (*p* = 0.080 and *p* = 0.097, respectively).

The rate of high eosinophilia (≥ 3.5%) was 74.5% in the intermittent group and 83.3% in the persistent group (*p* = 0.635). In the mild group it was 77.8%, and in the moderate/severe group it was 74.3% (*p* = 0.780). No significant difference was found in the four-subgroup comparison either (*p* = 0.927). Regarding symptom pattern, high eosinophilia was found in 70.8% of the seasonal group, 82.4% of the perennial group, and 75.0% of the undetermined group (*p* = 0.699).

## Discussion

There is no clear consensus among researchers regarding the use of biomarkers in the diagnosis of AR and NAR [17, 18]. Mixed rhinitis, in which allergic and non-allergic mechanisms coexist, can frequently lead to diagnostic and treatment errors. NC has enabled clear definition of NAR subtypes in its diagnosis as a response to non-specific stimuli such as temperature, humidity, and odor in the nasal mucosa [19, 20]. Although nasal smear eosinophilia is a supportive finding for AR, its routine use is limited due to the variability of its sensitivity and specificity. The nasal eosinophil ratio exceeds 20% in NARES (Nonallergic rhinitis with eosinophilia syndrome). Chronic eosinophilic inflammation is the main pathophysiological component of this group, a subtype of NAR, and studies in these cases have shown that the percentage of nasal eosinophils is significantly higher compared to control groups [21]. These findings support the notion that nasal eosinophilia is significantly higher in the NAR group compared to controls and that eosinophilic inflammation is the distinguishing characteristic of this syndrome. Similarly, other descriptive studies have used a nasal eosinophilia criterion of > 20–25% for defining NARES and have reported that this value is associated with the non-allergic form of chronic rhinitis [22]. In our study, we found that it was significantly higher compared to the control group. In our study, we counted 100 cells in the most dense cell region and determined the eosinophilia rate to be an average of 7% and a cut-off value of 3.5% in the NAR group. However, our nasal eosinophilia results were lower than in the literature. Furthermore, while the literature requires a high nasal eosinophil ratio for specific phenotypes, it is also emphasized that eosinophilic infiltration should be evaluated as a continuous spectrum within the broader NAR population, rather than as a strict threshold [22].

Factors such as inaccurate collection of nasal smears and improper storage conditions until microscopic examination may explain why our results were lower. Studies confirm that nasal cytology is a simple, non-invasive tool capable of identifying inflammatory involvement in non-allergic rhinitis, and show that diagnostic accuracy may be improved with samples taken from the middle meatus [23]. The middle meatus has been reported to perform better than inferior turbinate cytology in terms of the correlation between nasal and blood eosinophilia levels [24]. The middle meatus is the preferred site for nasal brushing as it provides information about viable epithelium and inflammatory cells [25]. Although we attempted to collect samples from the middle meatus in our study, patient compliance was not always optimal since the study group was pediatric. This may be another reason why our nasal eosinophilia values were lower than those in the literature. Our findings support that the assessment of nasal eosinophilia is a valuable biomarker for characterizing NAR subtypes; however, the effect of sampling site and technical standardization on results is significant.

Determination of the cut-off value for the nasal eosinophil ratio in the diagnosis provides standardization for interpreting results for researchers in future studies. For standardization, counting 10 different fields with the highest cell density has been recommended, but no consensus has been reported on whether only inflammatory cells or all cells should be counted [26]. The eosinophil percentage accepted as diagnostic is generally calculated among all cell types counted during the assessment. However, some researchers calculate the eosinophil ratio only among inflammatory cells [27, 28]. Because of this heterogeneity between studies, it has been reported that there is no single internationally accepted cut-off point [26]. Some researchers have reported that the lower limits for identifying an eosinophilic subphenotype in NAR are as low as 5% [29]. Ronchetti et al. reported that no eosinophils were detected in the majority of nasal smears from 183 unselected children aged 9–11 years, and when detected, the ratio was 1% or below [30]. In our study, this ratio was 7%. This result suggests an immune eosinophilic activation within the NAR-diagnosed group, albeit without a fully established clinical picture. The fact that the eosinophil levels detected in our study remained below the conventional thresholds in the literature can be attributed to several key factors. The reason for this lower result compared to the literature is thought to be the technical difficulty of obtaining swabs from the middle meatus in a pediatric population. We predict that the cut-off value may change as the number of patients increases. Furthermore, the majority of studies in the literature provide data regarding adult patients. However, the immature immune system in the pediatric population and the resulting dynamic nature of the nasal cytological profile suggest that these values may vary in children [31]. Another potential reason is that our study evaluated children with negative systemic atopy tests despite having allergic symptoms; the inability to utilize advanced techniques for differentiating LAR may have increased group heterogeneity and resulted in sub-threshold eosinophilia levels [32].

The clinical utility of the Nasal Allergen Provocation Test (NAPT), the gold standard for LAR diagnosis, remains limited in pediatric populations due to practical challenges, the lack of standardized protocols, and its invasive nature [33]. The absence of NAPT in our study introduces the possibility that patients with ‘Nasal Eosinophilia in Pediatric Rhinitis Without Systemic Atopy’ and those with LAR may have coexisted within our cohort. Nevertheless, the presence of eosinophilia detected via nasal cytology serves as a valuable indicator for understanding the local inflammatory phenotypes—either Nasal Eosinophilia in Pediatric Rhinitis Without Systemic Atopy or LAR—in these children without systemic atopy. Future studies incorporating large-scale NAPT and nasal-specific IgE measurements in the pediatric population would be appropriate to further clarify this distinction. However, the lower nasal eosinophil cut-off value compared to the literature, viewed from a different perspective, suggests that even lower levels of nasal eosinophil ratios may demonstrate clinical significance in the pediatric group and may allow us to capture patients more easily. Due to the invasive nature of the procedure in pediatric cases, patient compliance is often low, which complicates the collection of adequate cellular material from the nasal mucosa. This situation may have led to diluted results that could be defined as ‘physiological background noise’ in the eosinophil count [31]. The 3.5% ratio obtained in our study can be regarded as an ‘inflammatory signal’ in symptomatic children without systemic atopy. This rate may reflect a mild LAR phenotype—which could not be differentiated due to the aforementioned lack of allergen provocation tests—or non-specific mucosal irritation. We consider this value not as a strict diagnostic threshold, but as a preliminary finding indicating eosinophilic activity in non-atopic rhinitis in children.

Although family history of allergy or atopy has not been directly defined as a strong, consistent risk factor for NAR in studies examining the pathogenesis and risk factors of non-allergic rhinitis, the hypothesis of an underlying genetic predisposition is a strong one. A history of asthma and CRSwNP (Chronic Rhinosinusitis with Nasal Polyps) is frequently encountered in the family history of NAR patients [34, 35]. In a study of adults with allergic and NAR, a family history of allergic rhinitis was associated with symptom incidence in both groups, and family history was found to be linked to increased rhinitis symptoms even in the absence of atopy [36]. A study reported that the prevalence of family allergy history was at a similar level in both non-allergic rhinitis and allergic rhinitis groups, but stated that this did not indicate a genetic risk [34]. Another study found a significant association between a family history of nasal polyposis and NAR [34]. In general, epidemiological studies indicate that family history of allergic rhinitis may be associated with general chronic rhinitis symptoms. In our study, we also found a significantly higher rate family history of atopy in the NAR group.

ARIA is an international scientific initiative, classification system, and clinical guideline framework that systematically addresses the impact of AR (Allergic Rhinitis) on asthma [37]. It recommends classifying rhinitis based not only on allergic mechanisms but also on clinical severity and symptom duration; this classification is grounded in symptom continuity (intermittent vs. persistent) and severity (mild vs. moderate/severe) [37]. ARIA guidelines also provide direction on topics such as assessing the impact of allergic rhinitis on quality of life, pharmacological options, and joint management strategies with lower airway diseases [37]. The ARIA score has evolved into a multidimensional tool evaluating symptom severity, patient quality of life, and productivity loss. Furthermore, ARIA and VAS (Visual Analog Scale) scores have been shown to be reliable in reflecting clinical severity [38]. Demoly et al. reported that while ARIA and VAS scores show a strong correlation with AR symptom control and quality of life, this relationship is more limited in NAR [39]. In light of these data, it is understood that ARIA scoring provides more consistent results in assessing disease severity, quality of life, and treatment needs in AR, whereas in NAR, the clinical symptom pattern and response to external triggers should be primarily evaluated. The majority of the cases in our study consisted of intermittent (88.7%) and moderate-to-severe (66%) subjects. The groups exhibited a similar distribution in terms of nasal eosinophilia, and in contrast to the literature, no significant difference was identified between them. The PRQLQ was developed by Juniper et al. as a 23-item questionnaire to measure functional problems in rhinitis, such as symptoms, emotional functioning, and activity limitations [14, 40]. This test is a crucial assessment tool in rhinitis because the impact of the disease varies on an individual basis due to differences in personal perceptions of symptoms and disease severity [41]. Contrary to the existing literature, no significant correlation was found between PRQLQ scores and nasal eosinophilia in our study. The non-allergic inflammatory cascade and mucosal hyperreactivity observed in NAR may follow distinct patterns that do not always align with the chronicity and severity criteria established by ARIA. Consequently, ARIA might not be the optimal tool for determining the specific symptomatic burden or predicting the quality of life in NAR. Our findings demonstrate the necessity for developing disease-specific clinical scales and standardized scoring systems tailored specifically to the unique inflammatory landscape of NAR subtypes to achieve a more accurate clinical evaluation.

## Limitations

A major limitation of our study is the lack of Nasal Allergen Provocation Tests (NAPT) and local specific IgE measurements, which resulted in a heterogeneous study population potentially encompassing LAR phenotypes; this adds complexity to the interpretation of our findings. A secondary limitation is that the middle meatus, the most reliable sampling site for nasal cytology, could not always be fully sampled due to compliance issues in the pediatric patient group. Furthermore, the lack of technical standardization in storage conditions and slide preparation between sample collection and microscopic examination may have led to cell loss or a decline in staining quality, thereby affecting the results. The absence of a clear consensus in the literature on whether eosinophils should be calculated as a proportion of all cells or exclusively of inflammatory cells complicates the direct comparability of our study with existing research. Finally, the single-center design and the specific patient population of our study may limit the generalizability of the low cut-off values obtained to the broader pediatric population.

## Conclusions

In conclusion, although our study identified a significantly increased rate of nasal eosinophilia in pediatric NAR patients, our findings remain lower than the results typically reported in adult studies. These lower levels may reflect age-specific inflammatory responses or technical sampling challenges inherent to the pediatric population. The higher prevalence of a family history of atopy in the group with local eosinophilia suggests a complex genetic predisposition that warrants further investigation. Nasal cytology continues to be a valuable, non-invasive tool for initial screening; however, larger cohorts incorporating standardized sampling methods and NAPT are required to definitively differentiate these non-atopic eosinophilic phenotypes in children.

## Data Availability

The datasets used and/or analysed during the current study are available from the corresponding author on reasonable request.

## Abbreviations

ARIA: Allergic Rhinitis and its Impact on Asthma
AR: Allergic rhinitis
AUC: area under the curve
CRP: C-reactive protein
IgE: Immunoglobulin E
IQR: Interquartile range
LAR: Local allergic rhinitis
NAPT: Nasal Allergen Provocation Test
NAR: Non-allergic rhinitis syndrome
NC: Nasal cytology
PRQLQ: Paediatric Rhinitis Quality of Life Questionnaire
ROC: Receiver operating characteristic
SD: Standard deviation
VAS: Visual Analog Scale
